# Inhomogeneous Cortical Synchronization and Partial Epileptic Seizures

**DOI:** 10.3389/fneur.2014.00187

**Published:** 2014-09-24

**Authors:** Lorena Vega-Zelaya, Jesús Eduardo Pastor, Rafael G. de Sola, Guillermo J. Ortega

**Affiliations:** ^1^Clinical Neurophysiology Service, Hospital Universitario la Princesa, Madrid, Spain; ^2^Fundacion de Investigación Biomédica Hospital de la Princesa, Madrid, Spain; ^3^Neurosurgery Service, Hospital Universitario la Princesa, Madrid, Spain

**Keywords:** ECoG, synchronization, temporal lobe epilepsy, clusters

## Abstract

**Objective:** Interictal synchronization clusters have recently been described in several publications using diverse techniques, including neurophysiological recordings and fMRI, in patients suffering from epilepsy. However, little is known about the role of these hyper-synchronous areas during seizures. In this work, we report an analysis of synchronization clusters jointly with several network measures during seizure activity; we then discuss our findings in the context of prior literature.

**Methods:** Subdural activity was recorded by electrocorticography (with 60 electrodes placed at temporal and parietal lobe locations) in a patient with temporal lobe epilepsy with partial seizures with and without secondary generalization (SG). Both interictal and ictal activities (during four seizures) were investigated and characterized using local synchronization and complex network methodology. The modularity, density of links, average clustering coefficient, and average path lengths were calculated to obtain information about the dynamics of the global network. Functional connectivity changes during the seizures were compared with the time evolution of highly synchronized areas.

**Results:** Our findings reveal temporal changes in local synchronization areas during seizures and a tight relationship between the cortical locations of these areas and the patterns of their evolution over time. Seizure evolution and SG appear to be driven by two different underlying mechanisms.

## Introduction

In temporal lobe epilepsy (TLE), seizures are thought to originate in specific areas of the cortex, known as seizure-onset zones (SOZ), before spreading to other areas, known as epileptogenic zones (EZ), some of which overlap with the SOZ. EZ are essential for seizures to propagate (1). Resection or disconnection of these areas, principally of the EZ, which is usually identified as the epileptic focus, from the rest of the brain is currently considered the best way to eliminate seizures in drug-resistant TLE patients. This “single-focus” model has been challenged (2–4) in favor of a network model in which emphasis is shifted from the epileptic focus (or foci) toward the properties of the limbic network itself. Fortunately, in recent years, there have been great advances in the development of mathematical and numerical methods that aim to uncover the hidden properties of complex systems with mutually interacting parts. The introduction of the functional connectivity concept (5, 6) and the development of complex network methodology (7, 8) in recent years have not only provided analytical tools for exploring the physiology of the brain in general (9) and many of its pathologies, including epilepsy (10–15), but also allowed researchers to formulate new hypotheses about brain function/dysfunction. In the case of epilepsy, much work has focused on the properties of global and local cortical networks in recent years. Local synchronization (LS) and inhomogeneities in synchronization distribution, in particular, have received much attention (16–24). In these studies, the existence of an inhomogeneous distribution of functional connectivity among cortical areas in patients with TLE has been examined extensively. Although it is tempting to associate high LS with the traditional epileptic focus, whether a direct relationship can be established between them is currently unclear. However, these areas almost certainly play a role in seizure development because the excision of cortical areas that include high synchronization zones appears to abolish the appearance of seizures. Moreover, it has been suggested (18) and shown (23) that these areas also exhibit behavior that is very stable during the interictal stage. However, all previous work on LS areas has focused on the interictal period. To the best of our knowledge, no study has examined LS evolution during seizures, although many other network characteristics, such as centrality measures, have been examined during this critical period in epilepsy dynamics (10–13).

In this study, we investigated changes in high-LS areas during seizures, that is, the evolution of LS during the preictal, ictal, and postictal stages in such areas. We also calculated several standard global network measures. To generate as detailed an analysis as possible, we analyzed seizure evolution in a single patient suffering from TLE with partial seizures (PS) with secondary generalization (SG) and PS without SG. We present an analysis of subdural recordings of four seizures, three of them with SG and the fourth one without SG; studying both types of seizures in the same patient allowed us to discriminate between local and global seizure mechanisms.

Therefore, the aim of this paper is twofold. The first one is to show a detailed analysis in a single patient with several, although of different type, seizures, which otherwise may overlook important details in a more general and wide-ranging study. The second aim is to show the dynamical interplay between local and global network characteristics during the ictal stage.

## Materials and Methods

### Data

**Figure 1:**
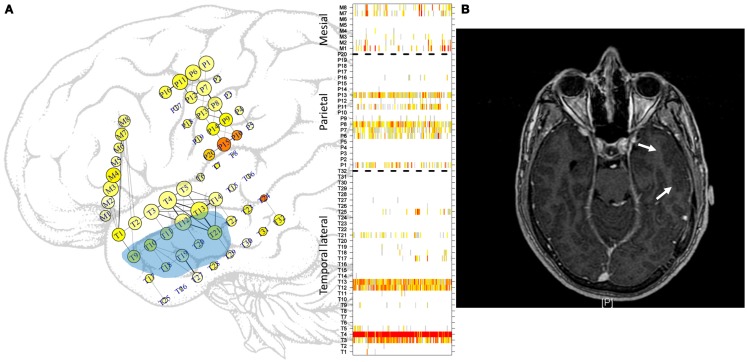
**Approximate locations of the subdural electrodes**. **(A)** Two electrode grids were deployed in the parietal (20 electrodes) and temporal (32 electrodes) lobes, and one mesial strip (8 electrodes) was also used. The location of the mesial strip is shown slightly incorrectly to enable clear visualization. Greater electrode sizes convey greater LS activity, and the colors convey the excitability at that location. Some of the more intense links between cortical areas are displayed. See the text for more details. Light blue area displays approximate location of gliosis. In the right side, ordering of electrodes in each sub-region, temporal lateral, parietal, and mesial, separated by dashed lines as used in subsequent figures displaying LS activity. **(B)** MRI image. Arrows mark the gliosis region.

Subdural recordings were obtained from a patient with refractory left TLE with PS and Chaslin gliosis (post-surgery anatomo-pathological assessment) ipsilateral to the seizure-onset area. Since the resective surgery, the patient has been in Engel’s grade IA (2 years). Three sets of electrodes were used. The study was approved by the Hospital Universitario de la Princesa ethics committee, and informed consent was obtained from the patient. One electrode grid was placed over the left parietal area (20 electrodes, P1–P20), and another electrode grid was placed over the lateral temporal lobe (32 electrodes, T1–T32). Additionally, a strip of eight electrodes (M1–M8) was placed over the mesial side of the temporal lobe, through the Sylvian fissure. Activity was then recorded from 60 electrodes and subsequently analyzed. Figure 1A shows a sketch of the approximate locations of the electrodes, although the mesial strip has been positioned incorrectly to facilitate visualization. Ordering of mesial (M1–M8), parietal (P1–P20), and temporal lateral (T1–T32) electrodes is also displayed at the right side of this figure. Main regions, as used in the following figures, are separated by horizontal dashed lines. The blue shadowed area displays the approximate gliosis region, marked by arrows in Figure 1B. A single reference electrode, located in the subgaleal area (not shown), was used for recordings. Electrocorticographic activity was sampled at 2 kHz, down-sampled at 200 Hz (Δ*t* = 0.05 between consecutive measurements) and then high-pass filtered at 0.5 Hz. A notch filter was used to eliminate the power-line frequency of 50 ± 0.25 Hz. Recordings made during interictal and ictal periods were saved for subsequent analysis. Interictal recordings that were made more than 1 h before seizures and that had no electrical artifacts (primarily due to patient movements) were selected for analysis. Based on these criteria, 1 h of interictal activity and four different seizures were selected for the present analysis. Data were analyzed in sliding 5-s epochs (containing 1000 data points per channel, sampled at 200 Hz), and identical numerical analyses (see below) were carried out for each epoch.

### Functional connectivity analysis and local synchronization

The functional connectivity between the time series of every pair of electrodes, *i* and *j*, was calculated using the absolute value of the Pearson correlation coefficient

(1) $$\rho_{\mathrm{ij}}=\left|\left.\frac{\sum_{\text{k}=1}^{N_{\text{win}}}\left(x_{i}\left(k\right)-\bar{x_{i}}\right)\left(x_{j}\left(k\right)-\bar{x_{j}}\right)}{\sqrt{\sum_{\text{k}=1}^{N_{\text{win}}}{\left(x_{i}\left(k\right)-\bar{x_{i}}\right)}^{2}\sum_{\text{k}=1}^{N_{\text{win}}}{\left(x_{j}\left(k\right)-\bar{x_{j}}\right)}^{2}}}\right|\right.$$

where ${\bar{x}}_{\text{i}}$ is the mean value of the time series *x*_i_ for channel *i* (which takes values from 1 to 60). Five-second epochs containing *N*_win_ = 1000 data points sampled at 200 Hz were used. Local synchronization (19) was estimated by averaging the correlations between each channel *i* with its first neighbors

(2) $$s_{i}=\frac{1}{n_{i}}\sum_{\text{j}=1}^{n_{\text{i}}}\rho_{\mathrm{ij}}$$

where *n*_i_ is the number of first neighbors (3, 5, or 8) for electrode *i* in a grid or in the strip (1 or 2). This “on-the-grid” representation provides us with an idea of the contribution of each cortical area to LS activity. To quantify the irritative and ictal activities in an objective way, we used the method of Bartolomei et al. (25) to calculate the measure

(3) $$S_{i}\left(k\right)=\left|\frac{x_{i}\left(k+1\right)-x_{i}\left(k\right)}{\Delta t}\right|$$

for each electrode-time series *x*_i_, where Δ*t* = 0.05 is the sampling time. Standard deviations of *S*_i_(*k*) were then calculated for the basal, i.e., interictal period, as $\sigma_{\text{i}}^{\text{ref}}=\sigma \;[S_{\text{i}}(k)]$ for each channel *i*. *S*_i_(*k*) was then normalized in such a way that ${\tilde{S}}_{i}=\frac{S_{i}\left(k\right)}{\sigma^{\mathrm{ref}}}.$ As in the work of Bartolomei et al., we find that epileptiform activity is present when ${\tilde{S}}_{i}>2.5$. This threshold value of 2.5 was empirically determined in Bartolomei et al. and works pretty well to define seizure activity in our own calculations. We used the first 160 epochs (approximately 13 min) of each interictal recording as the reference period. We call ${\tilde{S}}_{i}$ the excitability of channel *i* and refer to excitability generically to $\tilde{S}$.

A representation of both Eqs 1 and 2 is displayed on the grids in Figure 1A, in which LS is represented by the size of the electrode; greater electrode size corresponds to greater LS. In addition, ${\tilde{S}}_{i}$ is represented by the color of the electrode, ranging from white (no excitation) to red (high excitation). No relationship between the two measures exists. Some electrodes displayed high excitability with low LS, that is, with low levels of synchronization of the activity with its first neighbors. This phenomenon can be seen, for example, at electrode M7. The cortical area covered by this electrode displays irritative activity (orange color) but is actually disconnected from its first neighbor M8. In contrast, the area covered by electrode T4 is well connected with its neighbors but has rather low irritative activity. Another feature of Figure 1A is the representation of links between electrodes. A threshold of 0.5 (see Network Analysis below) has been used to represent functional connectivity between areas; i.e., only those areas having activities with a correlation coefficient (Eq. 1) greater than or equal to 0.5 are connected by straight lines. Moreover, the thicknesses of these lines are proportional to the strengths of these correlations.

**Figure 2:**
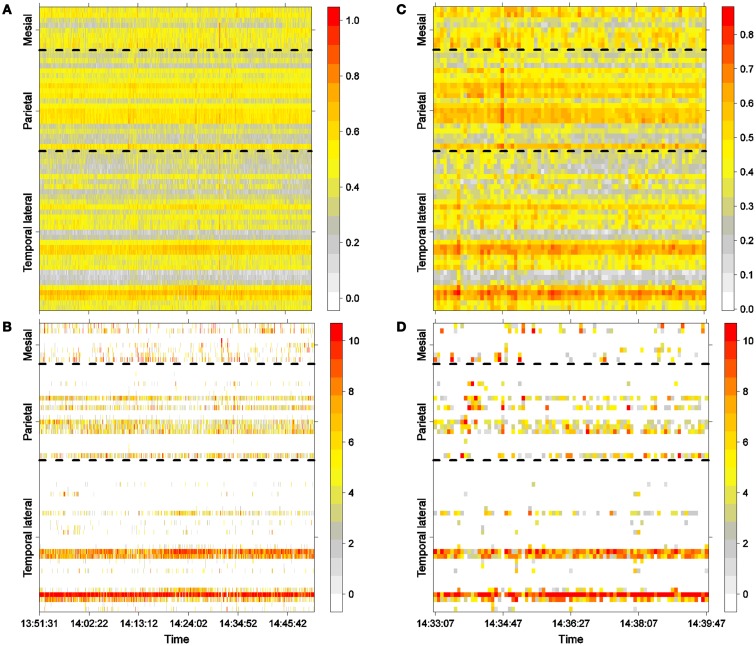
**Interictal activity**. **(A)** One hour of LS activity in each electrode (Eq. 2). **(B)** LS ordering. One hour of the 10 most active areas (greatest is 10). **(C)** Six minutes of LS activity in each electrode (Eq. 2). **(D)** LS ordering 6 min of the 10 most active areas (greatest is 10).

Figure 2A shows LS activity, as calculated using Eq. 2, during a 1-h interictal period. A stable, inhomogeneous pattern of LS appeared throughout the entire recording. To better display LS, we thus made one additional transformation; for each epoch, we organized the LS values in decreasing order of intensity and thereafter plotted only the first 10 most intense values, as shown in Figure 2B, in which red colors imply more intense LS activity (higher values). For example, areas covered by electrodes T4 and T13 exhibit higher LS.

### Network analysis

With the objective of evaluating the global aspects of cortical dynamics, we have calculated several of the most used measures of network properties. For this purpose, we used the R package igraph (26), which is widely used to calculate network properties. To reduce the number of edges in this fully connected network, we required that the intensity of the links be at least 0.5, i.e., an edge exists between nodes *i* and *j* only if ρ_ij_ ≥ 0.5 in Eq. 1. This particular threshold was chosen arbitrarily, with the objective of reducing the complexity of the network interactions to facilitate an exploratory study of the network dynamics. A more thorough study, beyond the scope of the present work, would require the network properties to be calculated for different threshold values.

The average path length and the density of edges were calculated for the entire 60-node network for each epoch. The average path length is calculated as the average of all the shortest paths, in terms of the number of steps along the network nodes, between every pair of vertices in the network. In this sense, low values for the average path length imply efficient and fast communication across the network functional topology. The density of the links is the ratio of the actual number of edges in the network to the number of all possible edges between the network nodes. A network with many links or a high density of links implies highly synchronous behavior at both short and long distances because more links are available when correlations are greater than the threshold used, which is, in this case, ρ_ij_ ≥ 0.5. Although a high density of links decreases the average path length in the network because a greater number of edges allows for a greater number of alternative paths between two nodes, the opposite is not always true, that is, a low average path length does not always imply a high density of links. A low average path length could also be obtained in a network with a few long-distance connections and a large number of local ones. The clustering coefficient characterizes local connectedness in a network by measuring how well-connected neighbors of a given node are. Thus, a low value of the average path length could also be obtained in a network with a low density of links, a high average clustering coefficient and a few long-distance links. This is the well-known small-world property of networks. An average clustering coefficient was also calculated for the whole network to explore this last possibility.

We also evaluated community structures in the whole network. A commonly used community-detection algorithm involving the maximization of modularity (27) was used. Modularity measures how well a given partition or division in a community in a complex network corresponds to a natural or expected sub-division. In our particular case, we have taken the “natural” community structure to be the one with three sub-networks corresponding to the parietal, lateral temporal, and mesial temporal electrode grids. Thus, the modularity of a network is a measure of how close the actual community structures calculated for each epoch are to the community structure composed of the three regional sub-networks.

The mean values (μ) and standard deviations (σ) of these four network measures were calculated during the interictal stage (first four rows in Table 1) to provide baseline values for the comparisons with these values during ictal activity (10). The percentage of the coefficient of variations (CV = σ/μ), also called the relative standard deviation, 100*CV, was used to determine the variability of these measures during the interictal period. The mean values of these network measures were used as baseline values for the comparisons of seizure data, as described below. Finally, average path length and clustering coefficient were calculated in random graphs during the interictal stage. The Erdös and Rényi model (8) was used in order to build random graphs with identical number of both nodes and links that the actual interictal network has in each temporal window. Mean value and standard deviation of these measures in random networks (Table 1) will help us to evaluate the small-world property in the functional network.

**Table 1:** **Four network measures calculated during the interictal stage: mean value (μ), standard deviation (σ) and percentage of relative standard deviation [(μ/σ) × 100] during 1 h of interictal stage**.

Network measure	μ	σ	(σ/μ) × 100
Modularity	0.46	0.05	10.32
Density	0.04	0.02	51.45
Average path length	2.63	0.48	18.35
Average clustering coefficient	0.59	0.07	11.86
Average path length (random graph)	4.22	0.75	17.77
Average clustering coefficient (random graph)	0.04	0.03	75

*Modularity (gray background) is a community structure measure. The last two rows correspond to average path length and clustering coefficient for interictal random graphs (see main text)*.

## Results

### Interictal analysis

One hour of interictal LS activity is represented in Figures 2A,B. In Figure 2A, LS activity is calculated using Eq. 2 for each electrode location. As shown previously (23), high values of LS are stable over time. In this particular case, areas near electrodes T4 and T12, which are spatially continuous and both located on the lateral side of the temporal lobe (see Figure 1A), have greater LS and more stable behavior as compared with other cortical areas. The LS values were arranged in descending order, and the 10 most intense nodes with high-LS values were plotted, as shown in Figure 2B. This latter figure confirms the existence of two areas of high LS near electrode T4 and another area near T12. Because these two electrodes are spatially contiguous, we may thus consider the region around both electrodes to be a single high-LS area. Other areas with high-LS values were also found in the parietal lobe, at electrodes P1, P6–P8, and P13. These electrodes are also spatially close and can be considered to be a single synchronization cluster. In the mesial area, several electrodes, most notably M1/M2 and M7/M8, exhibited high values of LS, but these LS were not stable over time. A closer look at interictal activity is shown in Figures 2C,D, where activity lasting approximately 6 min is expanded; both LS activity (Figure 2C) and LS ordering (Figure 2D) are shown.

To evaluate the global network properties, several network measures were calculated for the interictal period. The means and standard deviations of these network measures are shown in Table 1. The measure corresponding to the community structure, i.e., the modularity exhibited less variation than other measures, with relative variations during the interictal stage of 10.32. This measure yielded a value close to 0.5, meaning that intra-community links within the three principal brain regions, the parietal, lateral temporal, and mesial temporal areas, were as abundant as inter-community links. The densities of the links and the average path lengths were also calculated. The temporal stability of the density of the links was found to be weak, with this measure exhibiting a relative standard deviation of 50%. However, as will be observed below, its behavior is highly stable when comparing it during the ictal activity. Finally, an average clustering coefficient over all the nodes of the network was also calculated (8), hereinafter referred to simply as clustering coefficient.

A small-world behavior of the interictal network shows up when comparing against random networks with identical number of nodes and links as the originals (Table 1). During the interictal period, random networks have higher average path length – 4.22 in random networks and 2.63 in the interictal one – and much lower average clustering coefficient – 0.04 in random networks and 0.59 in the interictal one.

### Ictal analysis

**Figure 3:**
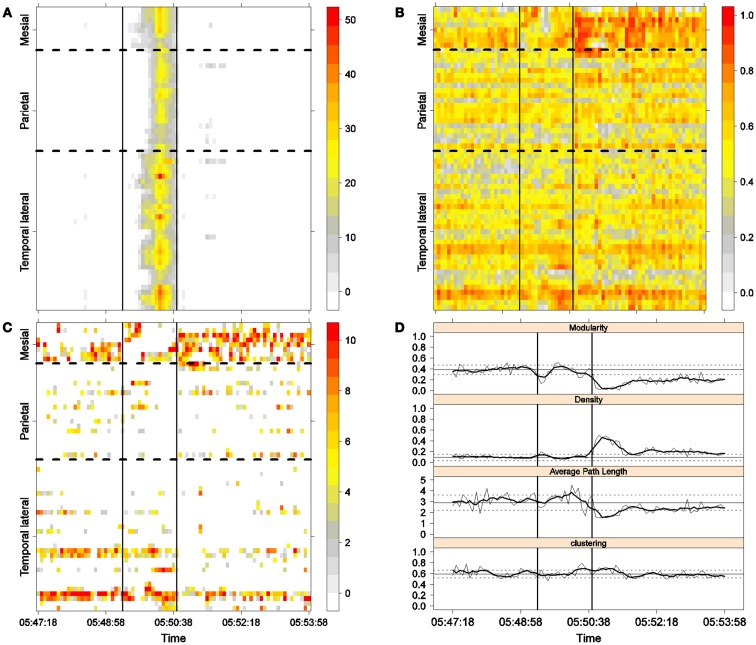
**First seizure**. Solid, vertical lines mark seizure onset and termination. **(A)** Excitability (Eq. 3). **(B)** LS activity (Eq. 2). **(C)** LS ordering. **(D)** Network measures. Horizontal solid and dotted lines show the mean and standard deviations of the corresponding measures during the interictal stage. The calculated values for each epoch are indicated by a thin, solid line, and the moving average of five consecutive epochs is shown with a thick, solid line.

**Figure 4:**
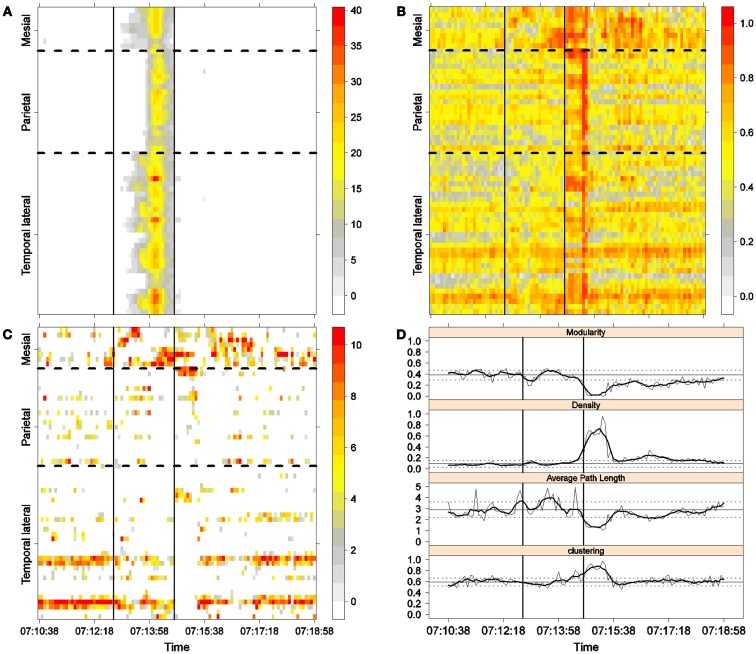
**Second seizure**. Solid, vertical lines mark seizure onset and termination. **(A)** Excitability (Eq. 3). **(B)** LS activity (Eq. 2). **(C)** LS ordering. **(D)** Network measures. Horizontal solid and dotted lines show the mean and standard deviations of the corresponding measures during the interictal stage. The calculated values for each epoch are indicated by a thin, solid line, and the moving average of five consecutive epochs is shown with a thick, solid line.

**Figure 5:**
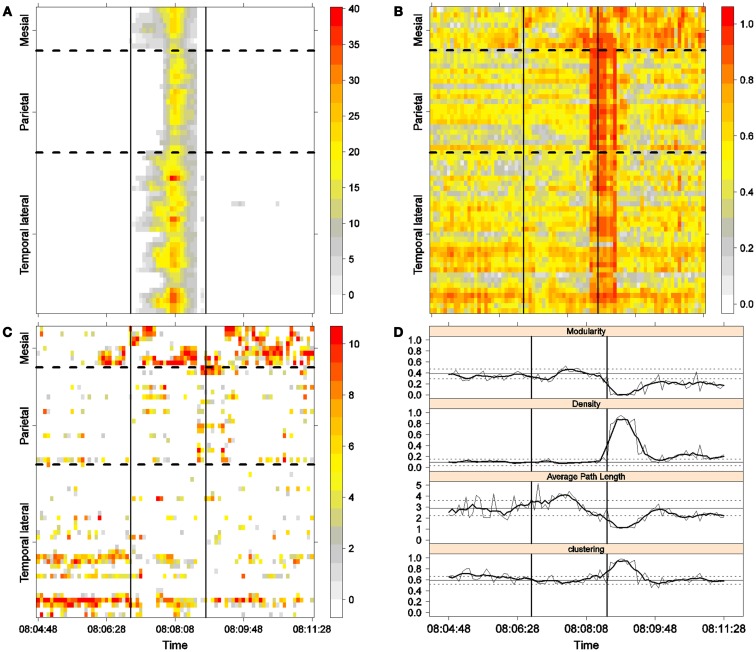
**Third seizure**. Solid, vertical lines mark seizure onset and termination. **(A)** Excitability (Eq. 3). **(B)** LS activity (Eq. 2). **(C)** LS ordering. **(D)** Network measures. Horizontal solid and dotted lines show the mean and standard deviations of the corresponding measures during the interictal stage. The calculated values for each epoch are indicated by a thin, solid line, and the moving average of five consecutive epochs is shown with a thick, solid line.

**Figure 6:**
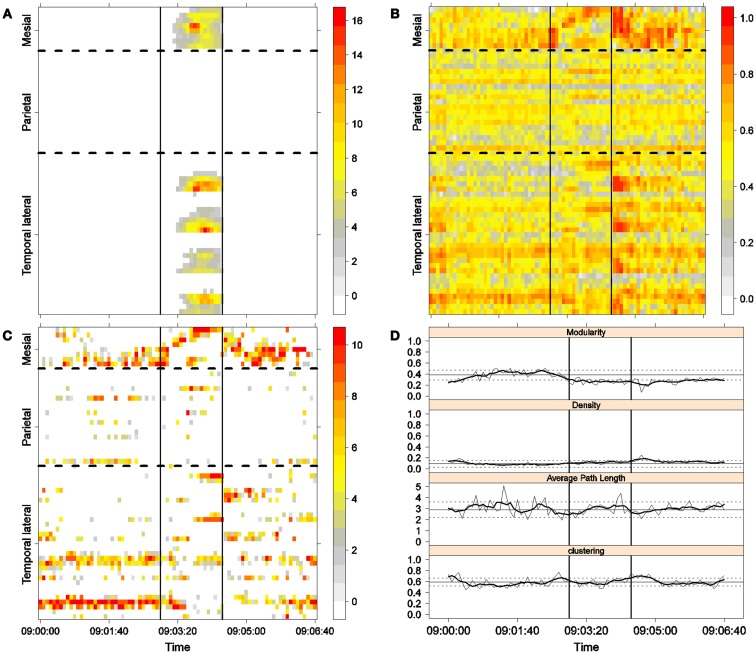
**Fourth seizure**. Solid, vertical lines mark seizure onset and termination. **(A)** Excitability (Eq. 3). **(B)** LS activity (Eq. 2). **(C)** LS ordering. **(D)** Network measures. Horizontal solid and dotted lines show the mean and standard deviations of the corresponding measures during the interictal stage. The calculated values for each epoch are indicated by a thin, solid line, and the moving average of five consecutive epochs is shown with a thick, solid line.

The ictal activity during seizures was evaluated, and the results are displayed in Figures 3–6. Seizure onset and termination, as determined by two neurophysiologists (LVZ and JP), are marked by vertical solid lines in each of these figures. The visual detection of seizure onset is a retrospective process, in the sense that the neurophysiologist first searched for ictal activity, which has the principal characteristic of faster, high-amplitude oscillations that appear simultaneously on several channels, and then looked back at the neurophysiological recording to determine at which point the epileptogenic activity actually began. In the cases shown here, seizure onset was determined by abrupt changes in the frequency domain instead of by changes in the signal amplitude. This fact explains why the seizure-onset times (vertical lines) appear to be distant from the high-amplitude ictal activity. Four seizures were analyzed. The first three correspond to PS with SG (Figures 3–5). The last one is a PS without SG (Figure 6). In each case, excitability (as quantified by Eq. 3), LS, LS ordering, and network measures were analyzed.

Excitability is displayed in panel (A) in each seizure, that is, Figures 3A, 4A, 5A, and 6A. The first three seizures correspond to a PS with onset in the mesial area and SG. In contrast, the last seizure, shown in Figure 6A is a PS without SG. The ictal patterns of the first three seizures are very similar with respect to both extent and amplitude. In the PS without SG, the ictal activity started in the mesial area and lateral temporal lobe but did not propagate to the parietal lobe. Moreover, its intensity was lower than that of the other three seizures. Although in all four cases, the seizures began slowly and unevenly, all of them terminated suddenly. Note that in all four cases, the seizure intensity reached its maximal value in approximately the second half of the seizure.

The LS activity is displayed in panel (B) in each figure. As above, the LS activity during the first three PS with SG is displayed in Figures 3B, 4B, and 5B. Figure 6B shows the LS activity during the PS without SG. By ordering the LS values by intensity, we obtain Figures 3C, 4C, 5C, and 6C. We observed similar patterns for each of the three cortical areas examined.

In the mesial area, two observations are noteworthy. First, immediately before seizure onset, high-LS activity was found near electrode M2, followed by a displacement of this activity toward other mesial electrodes. In each case, the pattern was very similar from M1/M2 toward M8. Although the interictal LS activity at electrodes M1/M2 was intense, as noted above, it was nonetheless intermittent during the interictal period, as evident in Figures 2B,D; however, all seizures appeared to begin with high LS near electrodes M1/M2. In contrast, the LS displacement from M1 to M8 appeared to occur during the first part of the seizure, at least in the first three seizures. In these cases, when high-amplitude oscillations began to appear [compare panels (A) and (C) in Figures 3–5] during the second half of the ictal activity, an abrupt jump in activity from M7/M8 back to M1/M2 was observed. This change was not the case for the PS without SG. In the PS without SG, the LS remained localized near M8 until seizure termination (Figure 6A).

As observed during the interictal periods, areas with high LS were observed, specifically near electrode P6 (Figure 2B). However, these areas of high LS lost their strength before, during, and after the seizure in every case.

On the lateral temporal side (T1–T32), the LS activity remained stable, at least during the first part of the seizure. A disruption occurred at approximately the middle of the ictal activity, during which no LS activity was observed at electrodes T4 and T12, but this LS activity was restored in the second half of the seizure, most notably at electrode T4. However, this restoration of LS activity near electrode T4 did not occur in the fourth seizure.

The following is a summary of our observations regarding the local activity during the seizures:

For PS with SG [panels (C) in Figures 3–5]:

(1) Mesial area: An increase in LS activity at seizure onset was observed at the lower mesial electrodes, M1/M2. The LS activity then shifted and moved along the other mesial electrodes until the middle of the seizure. A disruption of LS activity followed, with the activity moving from M7/M8 back to M1/M2.
(2) Lateral temporal area: Stable LS activity was observed until approximately the middle of the seizure. A disruption in the LS activity was then observed, which was followed by a restoration of LS, most notably at electrode T4, until seizure termination.

For PS without SG (Figure 6C):

(1) Mesial area: An increase in the LS activity at seizure onset was observed at the lower mesial electrodes, M1/M2. The LS activity then shifted and moved along the other mesial electrodes until the seizure ended.
(2) Lateral temporal area: Stable LS activity was observed until approximately the middle of the seizure. The LS activity was then disrupted until seizure termination.

Both types of seizures began in areas with high interictal LS. However, keeping in mind the above observations, PS without SG displays a pattern similar to the first parts of PS with SG. A central element during the onset of generalization appears to be the re-establishment of LS at both M1/M2 and T4, which is lacking in the seizure without SG. We will next explore this issue using global network measures.

To obtain a global picture of seizure dynamics, we studied four network properties. These results are displayed in panels (D) of Figures 3–6. A moving average across five epochs (thick solid lines) was used to smooth the results calculated for each individual epoch (thin solid line). In both of these figures, interictal mean and standard deviation values are plotted (solid and long, dashed, horizontal lines, respectively) for the calculated values.

The preictal measurements were similar in the four seizures, and differences between PS with and without SG appeared just after seizure onset. At that time, modularity, in the case of PS with SG, suffered a brief drop, but it recovered to preictal values by the middle of the seizure. In the case of PS without SG, the initial drop after seizure onset was instead maintained throughout the seizure period and continued into the postictal period, with no appreciable changes in other network measures. In contrast, all changes during PS with SG appeared during the middle of the seizure, when the average path length was maximized. In the later parts of the seizure, a clear trend toward the postictal period appeared in every measure, producing extreme values, such as the lowest modularity, lowest average path length, highest density, and highest clustering coefficient that were attained after seizure termination. Perhaps, the clearest picture of this trend can be seen in Figure 5D (third seizure), although similar features are replicated to varying extents in the other figures. These changes clearly imply that a “wave” of increasing global synchronization with a temporal origin in the middle of the seizure.

Two remarks are noteworthy about the above observations. The first is related to the increasing global synchronization originating during the middle of the seizure for the case of PS with SG (Figures 3D, 4D, and 5D). All changes in the global network measures that reached extreme values after seizure termination were initiated in the second part of the seizure. At that moment, however, most of the measures had attained interictal/preictal values, except for the average path length and perhaps modularity. The average path length increased from the time of seizure onset, without being accompanied by appreciable changes in other measures. The only explanation for this situation would be a change in the functional topology without any accompanying change in the global synchronization levels. The weak increase in modularity also supports this assumption and suggests that some inter-regional links may be converted into intra-regional ones. Note moreover that modularity reaches values close to 0.5, suggesting a highly modular network (8).

Second, the difference between the modularity behaviors just after seizure onset in two types of seizures is striking. A sustained decrease in modularity during PS without SG was the main characteristic of this type of seizure because no other changes occurred. This observation implies a reorganization of the network functional topology toward greater global connectedness without accompanying changes in global synchronization, i.e., toward more inter-regional and fewer intra-regional connections. These changes occurred immediately after seizure onset without SG, in contrast to the case of PS with SG. In PS with SG, the modularity underwent more changes throughout the course of the seizure.

The interictal small-world property behaves in a wandering fashion during the seizures extent. Although in the first part of the seizures, for the case of PS with SG, the average path length increases, making the network more regular, it then decreases during the second part of the seizures until the postictal period, with a simultaneous increase in the clustering coefficient, making thus the network more random. For the PS without SG, no appreciable change exists during the preictal, ictal, and postictal periods, maintaining, therefore, the interictal small-word characteristic.

## Discussion

In this study, we analyzed in detail both interictal and ictal activities, which were recorded with subdural electrodes, from the point of view of functional connectivity and network methodologies. Our results suggest that two mechanisms may be involved in seizure onset and evolution. The first mechanism is related to local activity. LS activity follows a stereotypical behavior from the start of a seizure and lasts until the middle of the seizure, regardless of whether SG occurs. If no SG appears, the seizure ends at that moment. In contrast, if SG develops, further LS behavior appears. During the second half of the seizure, high-LS areas are located at the same sites that exhibit high interictal LS. The second mechanism, inferred from the global network measures, appears just after seizure onset. From that moment forward, network dynamics follow two different routes. In PS without SG, the only appreciable change is decreasing modularity after seizure onset. In contrast, PS with SG displays a trend toward a generalized synchronization starting at the middle of the seizure, and the network parameters reach extreme values just after the seizure ends.

We suggest that the underlying mechanisms leading to SG appear to begin immediately after seizure onset because no appreciable differences in network status between PS with and without SG exist at seizure onset. From that moment forward, two different evolutionary paths are followed in each case. These different evolutionary paths do not, however, affect local dynamics because, in both types of seizure, the activity in high-LS regions evolves in the same stereotypical fashion until the middle of the seizure, when the ictal activity begins to spread to other cortical areas in the case of SG. By that time, not only is the network status different but the network parameters also continue to diverge until after seizure termination. Hence, we propose that two different mechanisms are responsible for the spread of seizures. These mechanisms are consistent with the findings of Milton et al. (28), who demonstrated that different routes with different propagation velocities are responsible for seizure spread.

From a seizure semiology perspective, the findings presented here have a clear meaning. Although there is no appreciable evidence of seizure anticipation from the functional network point of view, in line with recent findings (29), a suggestive change in network behavior show up just after seizure onset. This difference between PS with and without SG regarding modularity determines which type of seizure will develop. Local analysis based in LS display identical patterns in both kinds of seizures; however, global network findings show the existence of a differentiated behavior just after seizure onset and thus forecast a SG. Certainly, a central mechanism should be involved here. More difficult to explain is the potential relationship between the structural alterations in the cortical network due to the temporal gliosis found in this patient and the functional behavior. This alteration (Figure 1) is located in the lateral side of the temporal lobe and encompasses a small subset of temporal electrodes (T9–T13 and T18–T21). Whether a relationship between the high-LS area in electrodes T3–T4–T12–T3 and the gliosis area exists or not remains to be studied and evaluated.

In terms of functional network structure, the results presented here are not fully in agreement with the current knowledge. The small-world characteristic displayed during the interictal stage is in accordance with the more general literature of cortical functional networks (30) and of healthy subjects (31) but in contradiction with more specific studies (10) where a shift from a regular to a small-world architecture is reported during the preictal/ictal transition. The results presented here conversely are in accordance with an interictal small-world architecture with a slight shift toward a more regular network during the first part of the seizures with SG and a subsequent shift toward a more random network during the postictal period. This is not the case for PS without SG. In this last case, no appreciable changes occur whether in the ictal or in the postictal periods. In regard to seizures with SG, our findings would thus suggest a shift toward a more modular, instead of regular, network topology during the seizures, as compared with the interictal/preictal period. This change, however, is not observed in the case of the seizure without SG.

In regard to the postictal period, the results presented here are in agreement with current knowledge about seizure termination (11, 32). The abrupt increase in the density of links facilitates the correlations between distant areas and decreases the average path length. These changes could provide a basis for seizure termination through not an excitatory but rather by means of an inhibitory mechanism. This increase in correlations among brain regions after seizure termination has also been observed in several previous studies (10, 11, 32). However, the results presented here show that extreme values of density of links, average path length, and clustering coefficient are attained beyond seizure termination, although the underlying triggering mechanism begins during the seizure itself.

The analysis presented here suggests two types of therapeutic approaches: on the one hand, a local procedure, whether by a resective surgery or non-invasively by gamma-knife surgery (33), which eliminates interictal local synchronization areas. This procedure would provide a way to abolish the stereotyped LS spread during the first part of the seizures, both with and without SG, because, as we have shown above, the spread of LS areas always begins in the more intense LS regions. The second target for a therapeutic intervention would be trying to block the SG onset. SG begins with an increase in both the average path length and the modularity. This behavior does not occur during the seizure without SG. Thus, a possible anti-SG treatment could be the application of drugs that allow inter-region connections, that is, in the regions’ boundaries, increasing thus inter-regions’ connections and therefore blocking the increase in both the modularity and the average path length.

There are, however, specific limitations in the present work. The first one concerns the limited nature of the data. Subdural electrodes record cortical activity on the brain surface and thus capture only the horizontal propagation of ictal spread. Seizures are well known to propagate more slowly along intracortical paths than along vertical routes via myelinated axons. The analysis of subdural data provides only an incomplete picture of the features of seizure spread throughout the epileptic network, which comprises, in turn, many other cortical and subcortical structures. In this sense, one may consider the network analyzed in this work to be a projection of the actual epileptic network, and the results derived from the present analysis should be considered according to this perspective. For instance, differences between results based on the analysis of depth electrodes with the ones reported here could be explained by the above considerations. Second, the findings presented here (with *N* = 1) cannot be generalized (at least in a straight forward fashion) to other cases. Obviously, we do not expect that the same patterns and dynamics reported here will be present in other cases as for instance in extra-temporal seizures. As mentioned above, high-LS areas have been extensively described, as well as its relationship with seizures in TLE, but nothing is known, to the best of our knowledge, of its existence in other cases. This fact prevents any direct generalization of the results presented here in that direction. Even in the case of TLE, further work certainly is needed before general statements can be made. However, we feel that a pooled analysis of local and global network dynamics, as performed in the present work, may represent an optimal strategy for the investigation of ictal activity recordings of the great variety of epileptic disorders.

## Conflict of Interest Statement

The authors declare that the research was conducted in the absence of any commercial or financial relationships that could be construed as a potential conflict of interest.
